# Control of Parasitophorous Vacuole Expansion by *LYST/Beige* Restricts the Intracellular Growth of *Leishmania amazonensis*

**DOI:** 10.1371/journal.ppat.1000179

**Published:** 2008-10-17

**Authors:** Jude Wilson, Chau Huynh, Kathleen A. Kennedy, Diane M. Ward, Jerry Kaplan, Alan Aderem, Norma W. Andrews

**Affiliations:** 1 Section of Microbial Pathogenesis, Yale University School of Medicine, New Haven, Connecticut, United States of America; 2 Institute for Systems Biology, Seattle, Washington, United States of America; 3 Department of Pathology, University of Utah, Salt Lake City, Utah, United States of America; 4 Department of Cell Biology, Yale University School of Medicine, New Haven, Connecticut, United States of America; Washington University School of Medicine, United States of America

## Abstract

The intracellular protozoan *Leishmania* replicates in parasitophorous vacuoles (PV) that share many features with late endosomes/lysosomes. *L. amazonensis* PVs expand markedly during infections, but the impact of PV size on parasite intracellular survival is still unknown. Here we show that host cells infected with *L. amazonensis* upregulate transcription of *LYST/Beige*, which was previously shown to regulate lysosome size. Mutations in *LYST/Beige* caused further PV expansion and enhanced *L. amazonensis* replication. In contrast, *LYST/Beige* overexpression led to small PVs that did not sustain parasite growth. Treatment of *LYST/Beige* over-expressing cells with vacuolin-1 reversed this phenotype, expanding PVs and promoting parasite growth. The opposite was seen with E-64d, which reduced PV size in *LYST-Beige* mutant cells and inhibited *L. amazonensis* replication. Enlarged PVs appear to protect parasites from oxidative damage, since inhibition of nitric oxide synthase had no effect on *L. amazonensis* viability within large PVs, but enhanced their growth within *LYST/Beige*-induced small PVs. Thus, the upregulation of *LYST/Beige* in infected cells functions as a host innate response to limit parasite growth, by reducing PV volume and inhibiting intracellular survival.

## Author Summary

The protozoan parasite *Leishmania* causes serious infections in humans throughout the world. After being inoculated into the skin through the bite of infected sandflies, the parasites enter host cells and replicate. The lysosome-like intracellular vacuoles where *Leishmania amazonensis* replicates expand dramatically as the infection progresses. Here we studied the impact of vacuole expansion on the ability of the parasites to survive and replicate inside host cells. We found that the host cell responds to infection with *Leishmania amazonensis* by upregulating expression of *LYST/Beige*, a gene that regulates the size of lysosomes and of parasite-containing vacuoles. The parasites replicated more efficiently in the large vacuoles formed in cells that have a mutation in *LYST/Beige*, whereas the same cells overexpressing functional *LYST/Beige* generated small vacuoles that were not able to sustain parasite growth. Drug treatments that reduced or enhanced the size of parasite-containing vacuoles had a corresponding effect on intracellular replication, demonstrating that large vacuoles provide a growth advantage to *Leishmania amazonensis*. Our results indicate that host cells respond to *Leishmania* infections by producing a protein capable of reducing vacuole size, as a strategy to inhibit parasite growth.

## Introduction

Infections with the trypanosomatid protozoan *Leishmania* cause a broad spectrum of human diseases throughout the world. Depending on the parasite species, and on the genetic and immunological composition of the host, the clinical form can range from self-healing cutaneous lesions to severe visceralizing disease. The parasites enter mammalian hosts through the bite of sandflies, and replicate intracellularly as amastigotes. Although macrophages are considered the major host cell type for *Leishmania*, fibroblasts also harbor parasites and are thought to play an important role during latent infections [1]). In both macrophages and fibroblasts, intracellular amastigotes replicate within parasitophorous vacuoles (PV) that share several properties with late endosomes/lysosomes, including low luminal pH and the presence of lysosome-specific membrane proteins and acidic hydrolases [2],[3].

An important question in the study of *Leishmania* pathogenesis is how parasites persist indefinitely in the host, even after the development of immunity to reinfection [4]. Their exclusively intracellular life style suggests that amastigotes possess mechanisms to avoid killing by the abundant microbicidal products produced by activated host cells. The mechanisms of *in vivo* persistence are of particular interest in relation to *L. amazonensis*, a species that causes cutaneous leishmaniasis in the new world. Several lines of evidence indicate that *L. amazonensis* is particularly adept at surviving intracellular killing mechanisms, when compared to other *Leishmania* species [5]–[7]. Interestingly, the morphology of the PVs harboring *L. amazonensis* amastigotes (and other species from the *L. mexicana* complex) also differs dramatically from PVs containing other *Leishmania* species, such as *L. major* and *L. donovani*. Amastigotes of *L. mexicana* and *L. amazonensis* replicate within very large, communal PVs that continuously undergo fusion with lysosomes and phagolysosomes. In contrast, PVs containing *L. major* and *L. donovani* amastigotes partition as the parasites replicate, resulting in small compartments containing only one parasite per vacuole [2],[8]. It was suggested that PV expansion might protect *L. amazonensis* from host killing mechanisms, by diluting microbicidal molecules [9]. Here we directly investigated this hypothesis, by examining the expression pattern and role in infection of *LYST/Beige*, a gene known to regulate lysosome size in mammalian cells. Our results show that *L. amazonensis* infections upregulate *LYST/Beige* transcription, resulting in the control of PV expansion and inhibition of intracellular growth.

## Results/Discussion

### *LYST/Beige* mRNA transcription is upregulated in macrophages infected with *Leishmania*

Human mutations in *LYST* (also known as lysosomal trafficking regulator) are responsible for the Chediak-Higashi syndrome (CHS), an autosomal recessive disease characterized by severe immune deficiency, partial albinism and recurrent bacterial infections. Cells from CHS patients and their mouse counterparts, *beige*, have abnormally enlarged lysosomes and lysosome-related organelles [10]–[12]. LYST/Beige overexpression reduces the size of lysosomes, suggesting that this large cytosolic protein (∼430 kDa) is involved in regulating the size of these organelles [13]. Although the LYST/Beige mechanism of action is still unknown, yeast two-hybrid screens [14], expression of truncated constructs [15], and functional analysis of homologs containing similar BEACH domains [16],[17] suggest that it may provide an anchoring scaffold for kinases and other molecules controlling membrane fusion/fission reactions. To investigate a possible role of *LYST/Beige* in the regulation of *L. amazonensis* PVs, we initially focused our investigations on the expression levels of this gene in infected macrophages.

**Figure 1:**
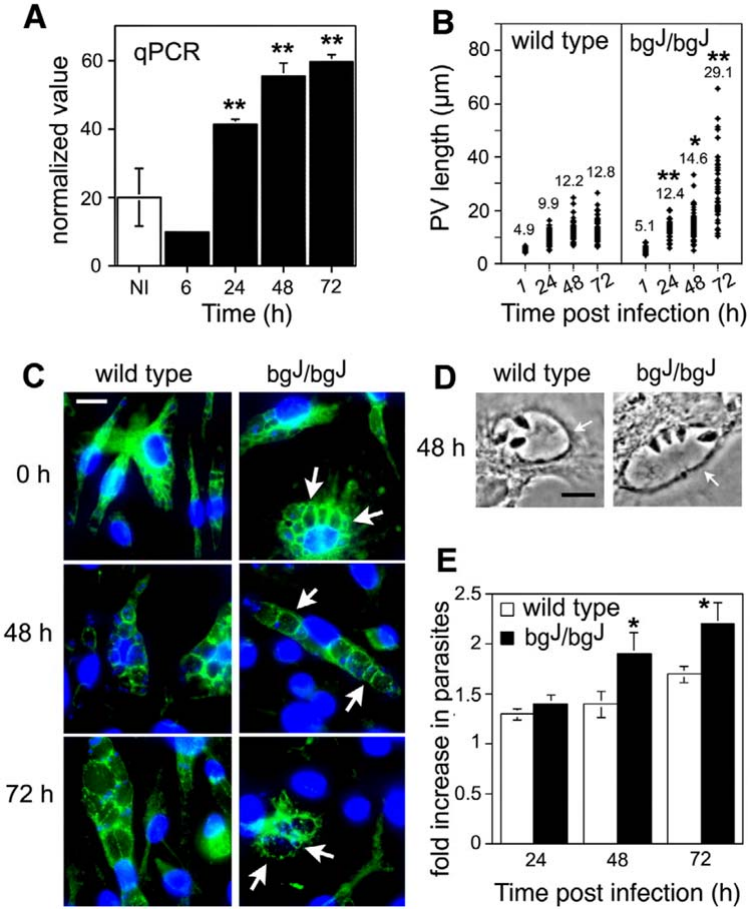
Upregulation of the lysosomal trafficking regulator gene (*LYST/Beige*) in macrophages infected with *Leishmania amazonensis* modulates parasitophorous vacuole expansion and parasite intracellular growth. (A) Upregulation of *LYST/Beige* transcripts in BMM infected with *L. amazonensis*. BMM were infected for 30 min with axenic amastigotes, washed and incubated for the indicated periods, followed by RNA extraction and quantitative real-time PCR (qPCR) analysis. The data represents the mean +/− SD of triplicate determinations. Asterisks indicate significant differences from non-infected samples (NI): ** *P*<0.005 (Student t test). (B) Increased PV expansion in *LYST/Beige*-deficient BMM (bg^J^/bg^J^). BMM were infected for 30 min with axenic amastigotes and the size of PVs was determined microscopically after the indicated periods. The values represent the mean of 50 independent PV measurements, asterisks indicate significant differences from the same time points in wild type BMM: * *P*<0.05, ** *P*<0.01 (Student t test). (C) Wild type and bg^J^/bg^J^ BMM infected with *L. amazonensis* were fixed immediately or after 48–72 h, and stained with anti-Lamp1 mAb (green) and DAPI (DNA, blue) stain. Arrows point to heterogeneously sized Lamp1-positive PVs in bg^J^/bg^J^ BMM. Bar = 15 µm. (D) Phase-contrast images showing higher magnifications of parasite-containing PVs (arrows) in wild type or bg^J^/bg^J^ BMM 48 h after infection. (Bar = 5 µm). (E) *L. amazonensis* intracellular growth is enhanced in bg^J^/bg^J^ BMM. BMM were infected for 60 min and the number of intracellular parasites was determined after the indicated periods. The data (expressed as fold increase in parasite numbers over the 60 min values) corresponds to the mean +/− SD of triplicates. Asterisks indicate significant differences from the corresponding time points in wild type BMM: * *P*<0.05 (Student t test).

Oligonucleotide DNA microarray analysis demonstrated that *LYST/Beige* transcription was increased in C57BL/6 mouse bone marrow macrophages (BMM) infected with *L. amazonensis* axenic amastigotes for 48 h (results not shown). These findings were confirmed using real time PCR (qPCR). Infection of BMM with *L. amazonensis* amastigotes induced a gradual enhancement in *LYST/Beige* messenger RNA transcription, reaching a ∼3 fold increase 72 h after infection (Figure 1A). As previously described for *L. amazonensis* [2], amastigotes replicating intracellularly during this period were visualized attached to the Lamp1-positive limiting membrane of large communal PVs (Figure 1B and 1C).

### The *Beige* mutation causes expansion of *Leishmania*-containing parasitophorous vacuoles and enhances parasite growth

To directly examine the role of LYST/Beige in controlling *L. amazonensis* PV expansion, we measured the size of parasite-containing vacuoles in BMM from wild type or *beige* mice (bg^J^/bg^J^). In wild type BMM infected with *L. amazonensis*, PVs expanded rapidly between 1 and 48 h after infection, and more slowly between 48 and 72 h (Figure 1B, wild type). In contrast, PV expansion in infected bg^J^/bg^J^ BMM appeared to be enhanced, with several significantly larger PVs observed at all time points (Figure 1B–1D, bg^J^/bg^J^). The PV size distribution in bg^J^/bg^J^ BMM, however, was heterogeneous (Figure 1B), with markedly enlarged PVs observed side-by-side smaller ones (arrows, Figure 1C). This pattern was consistent with the overall morphology of the lysosomal compartment in bg^J^/bg^J^ BMM, which is also heterogeneous in size (results not shown), probably due to the highly dynamic nature of the tubular lysosomes of macrophages [18]. When we analyzed the impact of LYST/Beige deficiency on *L. amazonensis* intracellular survival and growth in bg^J^/bg^J^ BMM, we found a small but statistically significant increase in the number of intracellular parasites at 48 and 72 h after infection (Figure 1E). Although the size heterogeneity of PVs in BMM did not allow a definitive conclusion, these data suggested that PV expansion might favor *L. amazonensis* survival and replication within host cells.

### LYST/Beige overexpression reduces parasitophorous vacuole size and inhibits parasite growth

**Figure 2:**
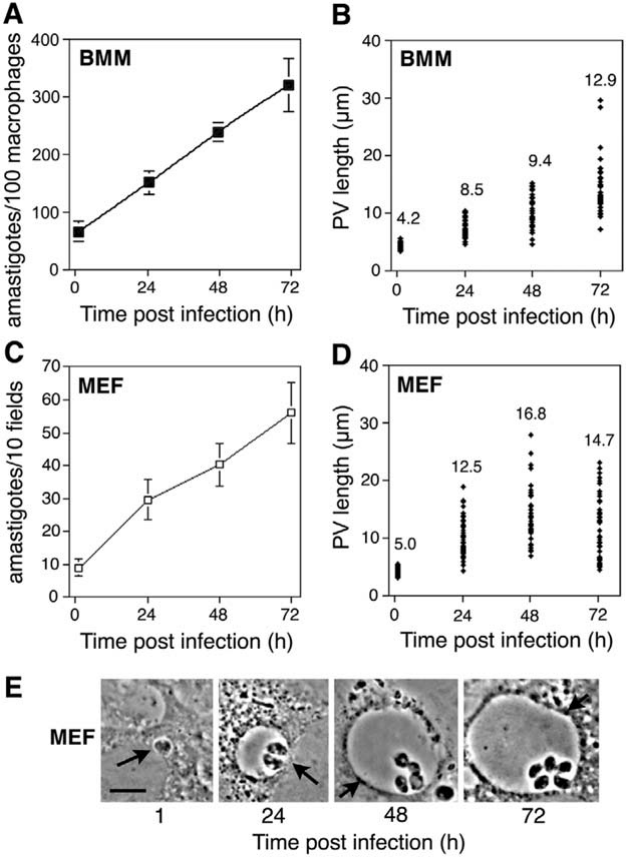
*L. amazonensis* parasitophorous vacuole expansion occurs faster in fibroblasts than in macrophages. (A,C) Intracellular growth of *L. amazonensis* amastigotes in mouse BMM (A) or MEF (embryonic fibroblasts) (C). BMM or MEFs were exposed to amastigotes at a MOI = 1 for 30 min or MOI = 10 for 60 min, respectively. Cells were washed, incubated for 1, 24, 48, and 72 h, and the number of intracellular parasites was determined. In fibroblasts parasites per microscopic field were quantified, to compensate for cell division during the experimental period. The data corresponds to the mean +/− SD of triplicates. (B,D) The average size of the parasitophorous vacuole (PV) in BMM (B) and MEFs (D) increases in parallel with the number of intracellular amastigotes. In MEFs the maximum PV expansion is reached earlier than in BMM. The values represent the mean of 50 independent measurements. (E) Phase contrast images illustrating the rapid expansion of a *L. amazonensis* PV in a fibroblast (arrows). Bar = 5 µm.

Fibroblasts have been implicated as important host cells during latent infections with *Leishmania* [1]. Consistent with this finding, primary and immortalized fibroblast cell lines are susceptible to *L. amazonensis* infection [3],[19]. Examining in parallel primary BMM and murine embryonic fibroblasts (MEF) infected with *L. amazonensis* axenic amastigotes, we found that both cell types sustain PV expansion and parasite intracellular proliferation (Figure 2A and 2D). We also observed that in fibroblasts PVs expand faster, and are significantly more homogeneous in size (Figure 2C and 2D, compare to Figure 2A and 2B). This finding allowed us to examine in detail the impact of LYST/Beige expression on *L. amazonensis* intracellular growth.

**Figure 3:**
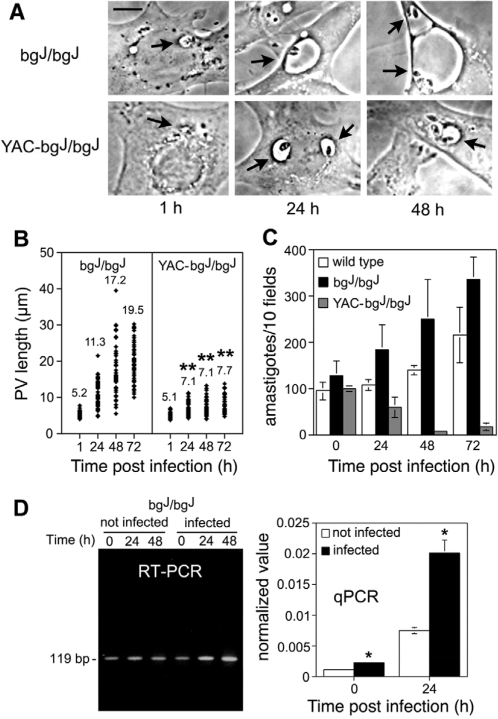
*LYST/Beige* overexpression in fibroblasts reduces parasitophorous vacuole size and inhibits parasite growth. (A) Phase contrast images showing that the PV expansion observed in bg^J^/bg^J^ fibroblasts is inhibited in YAC-bg^J^/bg^J^ fibroblasts overexpressing *LYST/Beige*. Arrows point to individual PVs. Bar = 10 µm. (B) Analysis of PV size in infected bg^J^/bg^J^ or YAC-bg^J^/bg^J^ fibroblasts, showing the markedly reduced PV expansion in cells overexpressing *LYST/Beige*. Fibroblasts were infected for 30 min, and PV size was determined microscopically after the indicated time points. The values represent the mean of 50 independent measurements. Asterisks indicate significant differences from the corresponding time points in bg^J^/bg^J^ fibroblasts: * *P*<0.05, ** *P*<0.01 (Student t test). (C) *L. amazonensis* amastigotes proliferate slowly in wild type and more vigorously in bg^J^/bg^J^ fibroblasts lacking a functional LYST/Beige protein, but not in YAC-bg^J^/bg^J^ fibroblasts that over-express *LYST/Beige*. Fibroblasts were exposed to *L. amazonensis* amastigotes for 60 min, washed, and incubated for the indicated periods followed by determination of the number of intracellular parasites. The data corresponds to the mean +/− SD of triplicates. The results shown are representative of several independent experiments. (D) Upregulation of the mutant *LYST/Beige* transcripts in bg^J^/bg^J^ fibroblasts infected with *L. amazonensis*. The cells were exposed for 30 min to axenic amastigotes, washed, and incubated for the indicated periods, followed by RNA extraction and analysis by qualitative RT-PCR (left) and quantitative real-time qPCR (right). The data on the right panel represents the mean +/− SD of triplicate determinations. Asterisks indicate significant differences from non-infected samples (NI): * *P*<0.05 (Student t test).

Fibroblasts derived from *beige* mice complemented (YAC-bg^J^/bg^J^) or not (bg^J^/bg^J^) with a wild copy of *LYST/Beige* [20] were infected with *L. amazonensis* axenic amastigotes. The size of *L. amazonensis*-containing PVs in the YAC-bg^J^/bg^J^ fibroblasts was markedly reduced, when compared to the PVs found in LYST/Beige-deficient bg^J^/bg^J^ cells (Figure 3A and 3B). Importantly, the overexpression of *LYST/Beige*, which is known to reduce lysosome size in this cell line [13], caused a reduction in PV size that was significantly below the size normally seen in *L. amazonensis*-infected wild type fibroblasts (compare Figure 3B with Figure 2D). This reduction in PV size had a strong impact on parasite growth, since no intracellular replication was detected in the YAC-bg^J^/bg^J^ cells overexpressing *LYST/Beige*, in several independent experiments (Figure 3C). A cell line derived in parallel from wild type mice also supported *L. amazonensis* intracellular replication, but the parasite population grew slower than in the mutant bg^J^/bg^J^ cells (Figure 3C). Collectively, these results support a role of *LYST/Beige* in restricting the intracellular survival of *L. amazonensis*.

We next examined whether the bg^J^/bg^J^ fibroblasts that sustain *L. amazonensis* growth also responded to infection by upregulating *LYST/Beige*, as seen in macrophages (Figure 1A). This determination was possible because the mutation causing *LYST/Beige* functional inactivation in bg^J^/bg^J^ fibroblasts would lead to a truncated protein, lacking approximately 1400 amino acids [20]. A single transcript corresponding to the truncated form of LYST/Beige was detected in bg^J^/bg^J^ fibroblasts (Figure 3D, left panel, not infected), and infection with *L. amazonensis* amastigotes markedly enhanced this signal (Figure 3D, left panel, infected, and Figure 3D, right panel). Thus, *L. amazonensis* infection triggers in host cells an innate response that upregulates *LYST/Beige* expression, a process that blocks parasite replication if the expressed protein is functional.

### The intracellular survival of *Leishmania* is regulated by parasitophorous vacuole size

**Figure 4:**
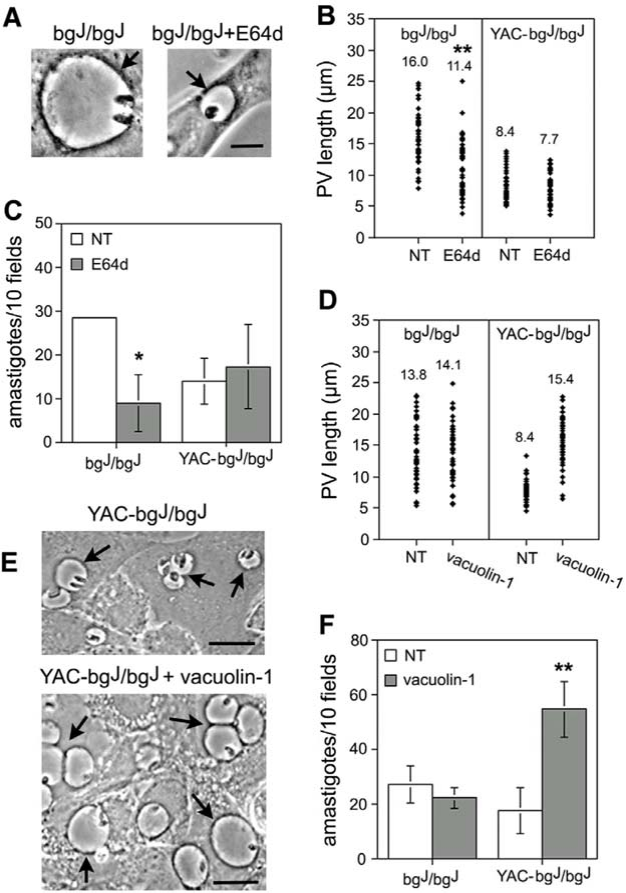
Parasitophorous vacuole size modulates the intracellular growth of *Leishmania*. (A) Phase contrast images showing that pretreatment with E64d reduces the large vacuoles observed in bg^J^/bg^J^ fibroblasts. Arrows point to individual PVs. Bar = 5 µm. (B) Analysis of PV size in infected bg^J^/bg^J^ or YAC-bg^J^/bg^J^ fibroblasts pretreated or not with E64d. Fibroblasts were exposed to *L. amazonensis* amastigotes for 30 min, washed, and further incubated for 24 h followed by microscopic determination of PV size. The values above the symbols represent the mean of 50 independent measurements. Asterisks indicate significant differences from the non-treated controls (NT): ** *P*<0.01 (Student t test). (C) PV reduction with E64d inhibits the intracellular growth of *L. amazonensis* in bg^J^/bg^J^ fibroblasts. After E64d pretreatment the fibroblasts were exposed to amastigotes for 1 h, washed, and further incubated for 24 h, followed by determination of the number of intracellular parasites. The data corresponds to the mean +/− SD of triplicates. Asterisks indicate significant differences from the non-treated controls (NT): * *P*<0.05 (Student t test). (D) Analysis of PV size in infected bg^J^/bg^J^ or YAC-bg^J^/bg^J^ fibroblasts pretreated or not with 1 µM vacuolin-1. Fibroblasts were exposed to *L. amazonensis* amastigotes for 30 min, washed, and further incubated for 24 h followed by microscopic determination of PV size. The values above the symbols represent the mean of 50 independent measurements. Asterisks indicate significant differences from non-treated controls (NT): ** *P*<0.01 (Student t test). (E) Phase contrast images showing that pretreatment with 1 µM vacuolin-1 enlarges the small vacuoles observed in *L. amazonensis*-infected YAC-bg^J^/bg^J^ fibroblasts. Arrows point to individual PVs. Bars = 10 µm. (F) After vacuolin-1 pretreatment the fibroblasts were infected for 1 h, washed, and further incubated for 24 h, followed by determination of the number of intracellular parasites. The data corresponds to the mean +/− SD of triplicates. Asterisks indicate significant differences from the non-treated controls (NT): ** *P*<0.01 (Student t test).

The data discussed above suggested that PV expansion facilitates the intracellular survival and growth of *Leishmania*. To further investigate this possibility, we examined the effects of reducing PV size in bg^J^/bg^J^ fibroblasts and, conversely, increasing the size of PVs in YAC-bg^J^/bg^J^ fibroblasts. Previous studies showed that treatment of bg^J^/bg^J^ cells with the thiol proteinase inhibitor E-64d reduces the size of the enlarged lysosomes normally observed in these cells [21],[22]. A reduction in PV size after pre-treatment with E-64d was evident in bg^J^/bg^J^ fibroblasts (Figure 4A and 4B), and this coincided with a reduction of ∼3 fold in the number of intracellular amastigotes detected after 24 h, when compared to untreated bg^J^/bg^J^ cells (Figure 4C, bg^J^/bg^J^). In contrast, there was no apparent effect of E-64d on the smaller PVs within the YAC-bg^J^/bg^J^ fibroblasts (Figure 4B, YAC-bg^J^/bg^J^), and no effect on parasite numbers in these cells (Figure 4C, YAC-bg^J^/bg^J^).

E-64d was washed out prior to infection, to prevent an effect of this irreversible cysteine protease inhibitor on the parasites. However, we cannot completely rule out the possibility that a small pool of the inhibitor remained inside host cells. For this reason, we examined the effect of a drug that has the opposite effect of E-64d, enlarging lysosomes. YAC-bg^J^/bg^J^ fibroblasts were pre-treated with vacuolin-1, a small molecule that causes rapid expansion of late endosomes/lysosomes in mammalian cells [23]. We infected bg^J^/bg^J^ and YAC-bg^J^/bg^J^ fibroblasts pre-treated with vacuolin-1 with *L. amazonensi*s, and determined PV size and the number of intracellular parasites after a 24 h period. Vacuolin-1 pre-treatment of bg^J^/bg^J^ fibroblasts did not further increase the mean PV size, nor did it cause an enhancement in the number of intracellular amastigotes (Figure 4D and 4F, bg^J^/bg^J^). In contrast, in the *LYST/Beige* overexpressing YAC-bg^J^/bg^J^ fibroblasts, vacuolin-1 caused a significant increase in PV size (Figure 4D–4E, YAC-bg^J^/bg^J^) and a ∼3 fold enhancement in parasite numbers (Figure 4F, YAC-bg^J^/bg^J^).

In these experiments, fibroblasts were pre-treated with E-64d or vacuolin-1 before infection to avoid any adverse effects on the parasites. For this reason, parasite development was not followed beyond 24 h. Since *Leishmania* has an intracellular doubling time of approximately 12 h, the strong impact of PV size on the number of parasites detected after only 24 h suggests that the mechanism involves direct modulation of early intracellular survival.

### PV enlargement may protect *Leishmania* from nitric oxide (NO)-mediated killing

A major mechanism underlying the leishmanicidal activity of murine macrophages is the IFN-gamma and TNF-alpha-mediated activation of nitric oxide synthase (iNOS or NOS2), which generates the potent microbicidal agent nitric oxide (NO) [24],[25]. Recent evidence indicates that iNOS is recruited to the membrane of recently formed pathogen-containing phagosomes, in a process thought to be critical for its microbicidal action [26],[27]. The strong reactivity of NO implies that it has to be generated in close proximity to targets, in order to be effective. Interestingly, *L. amazonensis* is significantly less susceptible to IFNgamma-mediated killing, when compared to species that reside in small PVs such as *L. major* [5],[6]. Thus, PV enlargement may have a role in reducing the concentration of NO in direct contact with intracellular amastigotes.

**Figure 5:**
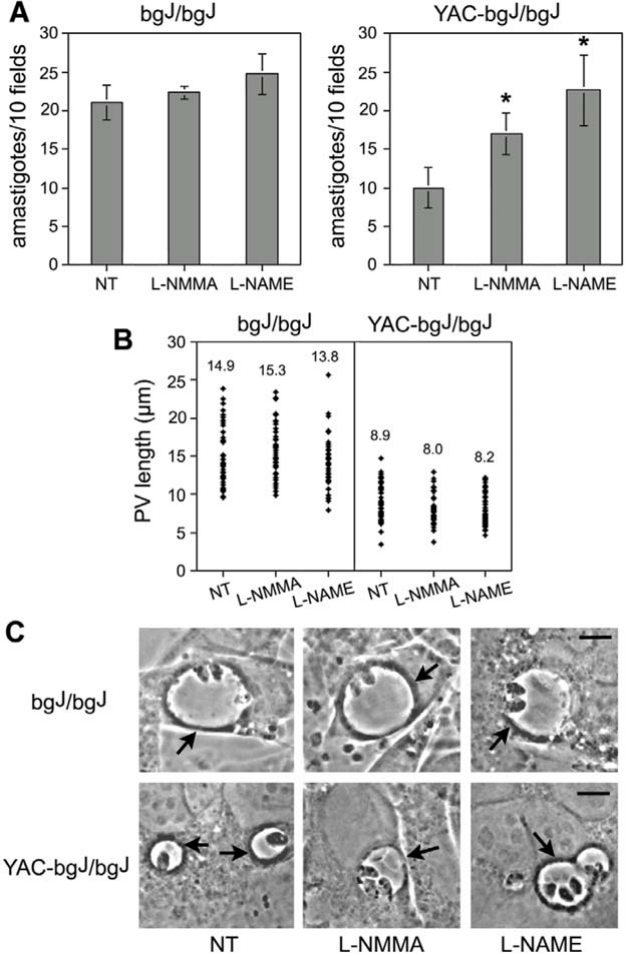
iNOS inhibition rescues *Leishmania* replication within small parasitophorous vacuoles. (A) Treatment with iNOS inhibitors does not affect *L. amazonensis* growth in the enlarged PVs of bg^J^/bg^J^ fibroblasts, but rescues growth in the small PVs of YAC-bg^J^/bg^J^ fibroblasts. After pretreatment with 50 µM L-NMMA or 50 µM L-NAME, the fibroblasts were infected for 1 h, washed and incubated for 24 h, followed by determination of the number of intracellular parasites. The data corresponds to the mean +/− SD of triplicates. Asterisks indicate significant differences from non-treated controls (NT): * *P*<0.05 (Student t test). (B) iNOS inhibitors do not alter PV size in bg^J^/bg^J^ or YAC-bg^J^/bg^J^ fibroblasts. Cells were exposed to *L. amazonensis* amastigotes for 30 min, washed, and further incubated for 24 h followed by microscopic determination of PV size. The values represent the mean of 50 independent measurements. (C) Phase contrast images of bg^J^/bg^J^ or YAC-bg^J^/bg^J^ fibroblasts pretreated or not with L-NMMA or L-NAME, exposed to *L. amazonensis* amastigotes for 1 h and fixed after 24 h. Arrows point to individual PVs. Bars = 5 µm.

Since *L. amazonensis* is capable of productively infecting both macrophages and fibroblasts in culture (Figure 2), and PV enlargement enhances the early survival and replication of *L. amazonensis* in these two cell types (Figures 3 and 4), it is conceivable that PV enlargement protects *Leishmania* from a microbicidal mechanism common to both macrophages and fibroblasts. Although NO production in response to IFN-gamma and TNF-alpha is much more robust in macrophages, fibroblasts are also capable of mounting this response [1]. To investigate whether NO production plays a role in the enhanced survival of *Leishmania* in fibroblasts, we quantified intracellular amastigotes in *L. amazonensis*-infected bg^J^/bg^J^ and YAC-bg^J^/bg^J^ fibroblasts pre-treated with the iNOS inhibitors L-NMMA and L-NAME. These inhibitors were previously shown to promote the intracellular growth of *Leishmania* in macrophages by blocking NO production [25],[28]. Quantification of the number of intracellular amastigotes after 24 h showed that these inhibitors did not alter the parasite population size in bgJ/bgJ fibroblasts, which exhibit large PVs (Figure 5A). However, amastigote survival and replication was significantly enhanced in YAC-bg^J^/bg^J^ fibroblasts pre-treated with the iNOS inhibitors (Figure 5B), consistent with the observation of several parasites inside each of the small PVs found in these cells (Figure 5D). Importantly, the size of the small PVs induced by *L. amazonensis* in the YAC-bg^J^/bg^J^ fibroblasts overexpressing *LYST/Beige* remained unchanged after treatment with the iNOS inhibitors (Figure 5C), suggesting that inhibition of NO production can promote *L. amazonensis* survival/growth in the absence of PV expansion. Future studies are required to investigate the possibility that *LYST* expression might also impact the trafficking of iNOS to *L. amazonensis*-containing PVs.

This study demonstrates that transcription of *LYST/Beige*, a gene previously implicated in regulating lysosome size, is upregulated in macrophages and fibroblasts infected by *L. amazonensis*. Examining the impact of PV expansion on the fate of the parasites, we found that PV size is an important determinant of intracellular survival. Collectively, our findings provide direct experimental evidence for the idea that increased expression of *LYST/Beige* functions as a host innate response to restrict *Leishmania* growth, by counteracting PV expansion. Our findings suggest that a search for drugs capable of specifically blocking *L. amazonensis* PV expansion might lead to novel therapeutic strategies against the human infections caused by this pathogen.

## Materials and Methods

### Parasites and mammalian cells

*Leishmania amazonensis* (IFLA/BR/67/PH8) were obtained from David Sacks (Laboratory of Parasitic Diseases, National Institutes of Health) and propagated as promastigotes at 27°C in M199 media supplemented with 5% penicillin/streptomycin, 0.1% hemin (25 mg/ml in 0.1N NaOH), 10 mM adenine, pH 7.5 and 10% FBS. To generate amastigotes, metacyclics were incubated in M199 media supplemented with 0.25% glucose, 0.5% trypticase, 40 mM sodium succinate (pH 5.4), 20% FBS, 5% penicillin/streptomycin at 2×10^6^/ml at 31°C for a minimum of 6 days, and passaged axenically at 31°C. All parasites were washed 3 times in PBS before use in experiments.

Bone marrow-derived macrophages (BMM) were isolated from the femurs of 8-10 week-old C57BL/6 wild type mice or beige mice (in the C57BL/6 background) and cultured for 7 days in RPMI 1640 containing 30% L-fibroblast culture supernatant and 20% FBS. Mouse murine embryonic fibroblasts (MEFs) were prepared from day 13.5 mouse embryos [29] and cultured in high glucose DMEM (GIBCO BRL) with 10% FBS, 1% penicillin/streptomycin, and 2 mM glutamine. Fibroblast lines from wild type C57BL/6J mice and from *beige-J* mice lacking the Beige gene (bg^J^/bg^J^) (in the C57BL/6J background), and the bg^J^/bg^J^ line complemented with a yeast artificial chromosome (YAC) carrying the Beige gene (YAC-bg^J^/bg^J^) (NCBI AAL40134 accession number) were generated in J.K.'s laboratory at the University of Utah [20] and maintained in DMEM 10% FBS, 1% penicillin/streptomycin and 2 mM glutamine (1 mg/ml G418 was kept in the growing medium of the *YAC-bg^J^*/*bg^J^* line, but removed prior to infection with *Leishmania*, followed by several washes).

### Affimetrix GeneChip analysis and quantitative real-time PCR

C57BL/6 BMM plated at 2.5×10^5^ in 10 cm dishes were exposed or not to *L. amazonensis* amastigotes at MOI∶10 for 30 min at 34°C. After infection cells were washed 3 times in PBS and further incubated for the appropriate times. Total RNA was isolated using Trizol (Invitrogen). The Affymetrix protocol, used to analyse RNA extracted from BMM infected for 48 h with *L. amazonensis* amastigotes, was essentially as described (GeneChip Expression Analysis Technical Manual, Affymetrix). cRNA was hybridized for 16 h to Affymetrix Genechip Mouse Genome 430 2.0 Array (Affymetrix), which contain 45,000 probe sets for the analysis of 39,000 transcripts and variants from over 34,000 mouse genes. Normalization was performed using GeneChip robust multi-array analysis, followed by GC-robust multi-array average (GC-RMA) normalization. Identification of significantly perturbed genes was done using significance analysis of microarrays. The false positive rate was 0.1%. Real-time PCR was performed using a BioRad iQ icycler Detection system (BioRad Laboratories, Ltd) with SYBR green fluorophore (BioRad Laboratories, Ltd) according to the manufacturer's instructions. Oligonucleotide primers 5′- AGCAGAAGGTGATAGACCAGAA and 5′-CCCACACTTGGATCATCAATGC were used to amplify a 119 base pair portion of LYST or 5′-TCAGTCAACGGGGGACATAAA and 5′-GGGGCTGTACTGCTTAACCAG to amplify a 142 base pair of the control cDNA HPRT1. The reaction was incubated for 3 min at 95°C, and then for 45 cycles of 20 s each at 95°C and for 1 min at 55°C. Fluorescence was detected at each annealing step and cycle threshold (*C*_t_) was calculated by determining the point at which the fluorescence exceeded a threshold limit. All reactions were run in triplicate and negative controls (no template cDNA) were included in each experiment. Data were normalized by the level of GAPDH expression in individual samples.

### Cell treatments

BMM were plated at 1.25×10^5^ and fibroblasts at 2.5×10^4^ on 12-mm diameter glass coverslips in 24-well dishes, 24 h prior to experiments. Vacuolin-1 (compound 5114069, ChemBridge San Diego, CA) (23), was added to fibroblasts at 1.0 µM for 1 h prior to infection. E-64d (Sigma) was added to fibroblasts at 1.0 µM for 1 h prior to infection. Immediately before infection the cells were washed 3 times in PBS. *N*-methyl-L-arginine (L-NMMA) or *N*-nitro-L-arginine methyl ester (L-NAME) (Sigma), two nitric oxide synthase inhibitors [26],[30], were added to cells at 50 µM for 1 h. Cells were washed in PBS prior to infection.

### *Leishmania* infection assays

For invasion of BMM, purified axenic amastigotes were added at a multiplicity of infection (MOI)∶1 in RPMI 10% FBS for 1 h at 34°C. For invasion of fibroblasts, amastigotes were added at an MOI = 10 for 1 h in high glucose DMEM supplemented with 10% FBS at 34°C. After invasion, cells were washed 3 times in PBS and incubated for indicated times at the appropriate temperature. Coverslips were then fixed in 2% paraformaldehyde, and host cell and parasite DNA were stained with 10 µg/ml DAPI for 5 min, after permeabilization with 0.05% Triton-X 100 for 10 min. At least 300 host cells, in triplicate, were analyzed for each time point. Images were acquired through a 100× objective using a Zeiss Axiovert microscope equipped with a Hamamatsu Orca II cooled CCD camera controlled by Metamorph Software (Molecular Devices). For parasite growth curves in fibroblasts the data was expressed as intracellular parasites per microscopic field, to compensate for host cell division [16]. For Lamp-1 immunofluorescence, cells on coverslips were permeabilized with PBS/ 2% BSA/ 0.5% saponin for 20 min, incubated with anti-Lamp1 mAb 1D4B (Developmental Studies Hybridoma Bank) 1∶50 in PBS/2% BSA/0.5% saponin for 45 min, followed by Alexa 488- goat anti-rat secondary antibodies (Invitrogen). Host and parasite DNA were stained with DAPI. Individual parasitophorous vacuoles (PVs) were measured using the Metamorph draw function. A line was drawn on phase contrast images straight across the length of the PV, and the Metamorph calculate function was used to convert the values to µm. A total of 50 PVs were measured for each sample.
